# Single-crystal-to-single-crystal phase transitions of commensurately modulated sodium saccharinate 1.875-hydrate

**DOI:** 10.1107/S2052252520015912

**Published:** 2021-01-01

**Authors:** Toms Rekis, Achim M. Schaller, Surya Rohith Kotla, Andreas Schönleber, Leila Noohinejad, Martin Tolkiehn, Carsten Paulmann, Sander van Smaalen

**Affiliations:** a Laboratory of Crystallography, University of Bayreuth, 95447 Bayreuth, Germany; b P24, PETRA III, Deutsches Elektronen-Synchrotron, 22607 Hamburg, Germany; c Mineralogisch-Petrographisches Institut, Universität Hamburg, 20146 Hamburg, Germany

**Keywords:** phase transitions, molecular crystals, aperiodic structures

## Abstract

This work reports reversible, single-crystal-to-single-crystal phase transitions of commensurately modulated sodium saccharinate 1.875-hydrate [Na(sac)(15/8)H_2_O]. The phases were studied in the temperature range 298 to 20 K. They exhibit complex disordered states. An unusual reentrant disorder has been discovered upon cooling through a phase transition at 120 K. The disordered region involves three sodium cations, four water molecules and one saccharinate anion. At room temperature, the structure is an eightfold superstructure that can be described by the superspace group *C*2/*c*(0σ_2_0)*s*0 with **q** = (0, 3/4, 0). It demonstrates maximum disorder with the disordered chemical entities having slightly different but close to 0.50:0.50 disorder component ratios. Upon cooling, the crystal tends to an ordered state, smoothly reaching a unified disorder component ratio of around 0.90:0.10 for each of the entities. Between 130 and 120 K a phase transition occurs involving a sudden increase of the disorder towards the disorder component ratio 0.65:0.35. Meanwhile, the space group and general organization of the structure are retained. Between 60 and 40 K there is another phase transition leading to a twinned triclinic phase. After heating the crystal back to room temperature its structure is the same as before cooling, indicating a complete reversibility of the phase transitions.

## Abstract 2

Two phase transitions of commensurately modulated Na(sac)(15/8)H_2_O have been discovered upon cooling. All three phases involve complex disorder. The crystal exhibits an unusual reentrant disorder upon cooling through the first phase transition at 130 K.

## 1. Introduction   

The saccharin molecule (sac) is known to be a versatile ligand for metal complex formation (Baran, 2005 ▸; Arslan Biçer *et al.*, 2017 ▸; Baran & Yilmaz, 2006 ▸). Its sodium salt has been used in the food industry as artificial sweetener for decades (Baran & Yilmaz, 2006 ▸). The crystal structure of sodium saccharinate hydrate has been shown to feature a large unit cell with *Z*′ = 16 formula units in the asymmetric unit (space group *P*2_1_/*n*). Furthermore, it has an unusual stoichiometry regarding water molecules – Na(sac)(15/8)H_2_O (Naumov *et al.*, 2005 ▸; Banerjee *et al.*, 2005 ▸).

So-called high-*Z*′ structures (*Z*′ ≥ 4) containing *Z*′-symmetry-independent copies of a single molecule are rare and still puzzle crystallographers, especially since many of them display pseudo-symmetries (Brock, 2016 ▸; Desiraju, 2007 ▸; Steed, 2003 ▸; Steed & Steed, 2015 ▸; Doboszewski *et al.*, 2018 ▸; Ganadu *et al.*, 2018 ▸; Brock & Taylor, 2020 ▸). This is also the case for the title compound. In a recent study, we have shown that this high-*Z*′ structure can be preferably described as a commensurately modulated phase in (3+1)-dimensional superspace [superspace group *C*2/*c*(0σ_2_0)*s*0, **q** = (0, 3/4, 0)] (Rekis *et al.*, 2020 ▸). The superspace approach rationalizes the origin of the high-*Z*′ phase as it allows a reduction in *Z*′ from 16 to 2 in a 4× smaller unit cell with an additional lattice centering.

Modulated structures of organic compounds are not widely explored, especially incommensurately modulated ones (Schönleber, 2011 ▸). For example, in the case of the pharmaceutical ciclopirox the diffraction data clearly indicate several incommensurately modulated phases (Brandel *et al.*, 2015 ▸). However, a structure model is only presented for the commensurate phase (as a high-*Z*′ structure in a supercell). It is believed that many other high-*Z*′ structures are actually modulated structures (Brock, 2016 ▸) and could be better described using the superspace approach. It is common that a high-*Z*′ (modulated) phase is related to a low-*Z*′ phase. Usually, at high temperatures a low-*Z*′ structure of higher symmetry exists that transforms into a pseudosymmetric high-*Z*′ phase upon cooling. For example, *p*-chlorobenzamide exhibits a *Z*′ = 1 phase at higher temperatures and a commensurately modulated phase (*Z*′ = 3 in 3D space) at lower temperatures (Schönleber *et al.*, 2003 ▸). Similarly, [(*tert*-butyldimethylsilyl)oxy]-4-ethynyl-4-(1-fluorohexyl)-1,2,3-oxa­thiazocane-2,2-dione exhibits a disordered *Z*′ = 1 phase at 200 K and an isostructural *Z*′ = 12 phase at 100 K (Vinokur *et al.*, 2017 ▸). Another example is 6-chloro-4,4,5,7-tetramethyldihydrocumarin which has a *Z*′ = 4 phase at normal pressure and a structurally similar *Z*′ = 2 phase at elevated pressure (Rajewski *et al.*, 2018 ▸). The description of such high-*Z*′ phases in superspace is highly recommended as some might even turn out to be incommensurately modulated, meaning that the 3D superstructure is only an approximation. It has been shown that reasonable supercell approximations of incommensurate phases can be obtained in 3D space (Schönleber & Chapuis, 2004 ▸); however, depending on the modulation wavevector **q**, such structure models can be unsatisfactory for detailed analysis of intermolecular interactions. The superspace approach is far more superior, even when dealing with commensurately modulated structures allowing us to rationalize phase transitions and material properties in terms of structural modulations (Pinheiro & Abakumov, 2015 ▸). High-*Z*′ phases and their possible modulations deserve comprehensive crystallographic studies to better understand molecular and metal–organic crystals and to be able to take such phases into account during crystal structure predictions, which is a crucial field in the development of solid-state chemistry and physics (Price, 2018 ▸).

**Figure 1:**
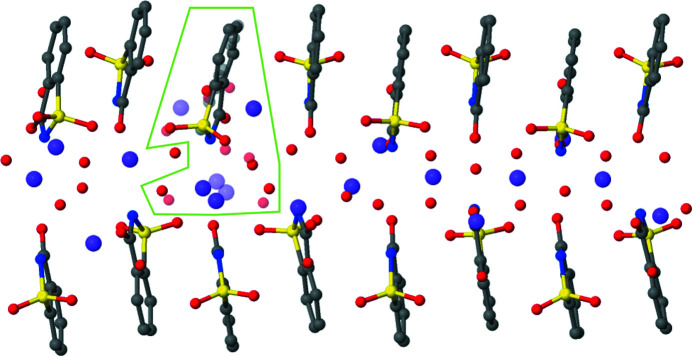
Asymmetric unit of sodium saccharinate 1.875-hydrate (for the 3D supercell in *P*2_1_/*n*) with the disordered region indicated. Minor components of the disordered sites are depicted as partly transparent symbols. Adapted from the work by Rekis *et al.* (2020 ▸).

As mentioned, the structure of Na(sac)(15/8)H_2_O was found to exhibit a commensurate modulation (Rekis *et al.*, 2020 ▸). However, the complexity of this structure can still be regarded as remarkable. The saccharinate anions demonstrate a simple displacive modulation throughout the unit cell. The modulation also includes some minor variation of the molecular conformations. However, only part of the sodium cations and water molecules can be considered modulated in a similar way. The remaining sodium cations and water molecules do not follow the main organizational pattern of the structure. Furthermore, most of the oddly positioned atoms are each disordered over two sites. This includes (in the *Z*′ = 16 supercell) three sodium cations, four water molecules and one saccharinate anion. In Fig. 1 ▸ it is shown how the smooth positional modulation in Na(sac)(15/8)H_2_O is interrupted by the complex disorder entity. The observed disorder seems to be an intrinsic property of this material, since similar geometry of the disorder components was noted in three independent diffraction experiments of this compound (Naumov *et al.*, 2005 ▸; Banerjee *et al.*, 2005 ▸; Rekis *et al.*, 2020 ▸). Any non-equilibrium crystallization effects or subtle influence of the crystallization conditions can therefore be excluded as the origin of the observed disorder.

**Figure 2:**
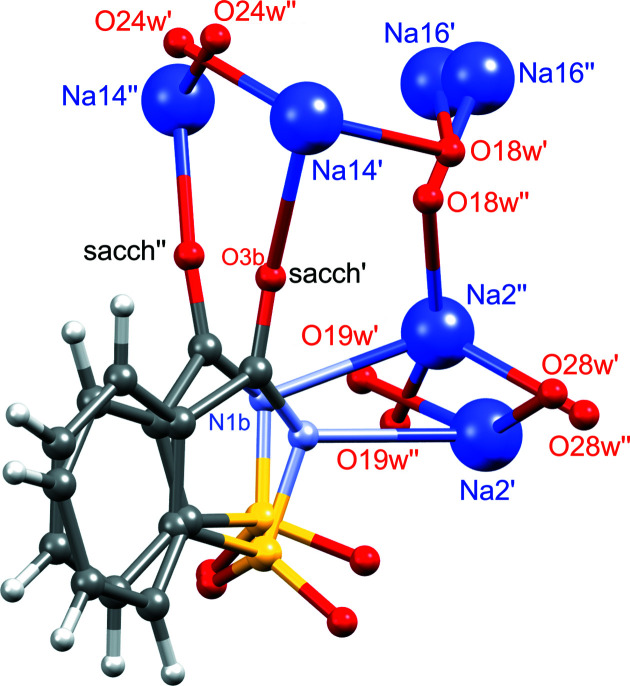
Disordered chemical entities in sodium saccharinate 1.875-hydrate (298 K model). Bonds between the entities are drawn according to the correlated disorder assumption showing two alternative realizations of the structure.

In previous studies, the disorder was treated as correlated, for example, two unified disorder components were considered where all the distinct chemical entities form one or the other ensemble. Disordered chemical entities are depicted in Fig. 2 ▸. Bonds between the entities are drawn according to this correlated disorder assumption. In our previous publication (Rekis *et al.*, 2020 ▸), we showed that separate refinement of the occupancy ratios of each pair of disordered chemical entities led to a single ratio of approximately 0.88:0.12 (single-primed:double-primed components), and eventually only one occupancy parameter could be used for the refinement. For the two structure models in the earlier literature, a single-occupancy ratio was reported of approximately 0.70:0.30 (Naumov *et al.*, 2005 ▸; Banerjee *et al.*, 2005 ▸). All crystals have been measured at 95 or 100 K. This discrepancy in disorder ratios is clearly a subject for future research.

The aim of the present study is to explore the nature of the disorder present in Na(sac)(15/8)H_2_O and to search for a possible optimal packing (no disorder) at low temperatures. That includes structure determinations at very low temperatures where disorder usually ceases or ‘freezes’ upon a glass transition. It was found that the occupancy ratio is temperature dependent and two phase transitions occur upon cooling.

## 2. Experimental   

### 2.1. Materials   

Sodium saccharinate hydrate (assay ≥ 99%) was purchased from Sigma–Aldrich and used without further purification. Deionized water was treated in a Millipore purification system. Single crystals were grown by slow evaporation from water solution. Transparent, prismatic crystals of different sizes were obtained. A crystal 0.15 × 0.15 × 0.15 mm in size was used for the diffraction experiment.

### 2.2. X-ray diffraction data collection   

Diffraction data were collected at beamline P24 of PETRA-III at the German Electron Synchrotron (DESY) facility. A wavelength of 0.5000 Å was used. The data were recorded on a Pilatus 1M CdTe detector. In total, six runs (ϕ-scans) were measured at each temperature with the following parameters: (1) attenuation factor (*att*) of 1, 2Θ_offset_ = 0°; (2) *att* = 1, 2Θ_offset_ = 7°; (3) *att* = 10.54, 2Θ_offset_ = 0°; (4) *att* = 10.54, 2Θ_offset_ = 7°; (5) *att* = 111.2, 2Θ_offset_ = 0°; 6) *att* = 111.2, 2Θ_offset_ = 7°. For each run, 3620 frames were collected with an exposure time of 0.1 s per 0.1°. Each consecutive 10 frames were subsequently binned using the *Pylatus* software (Dyadkin *et al.*, 2016 ▸) to obtain 362 frames of 0.1° per frame.

Datasets were collected at several temperatures maintained using an open-flow helium cryostat CRYOCOOL. A cooling rate of 4 K min^−1^ was used. The temperature profile of the measured single crystal was as follows: 298, 270, 250, 210, 170, 140, 130, 120, 110, 100, 90, 80, 70, 60, 40, 20 K. The crystal was then heated to 298 K at a heating rate of 8 K min^−1^. After around 15 h the same crystal was once again measured at 298 K.

### 2.3. Data integration   

The *EVAL14* software suite (Duisenberg *et al.*, 2003 ▸) was used for data integration. In the case of twinning (*T* = 20 and 40 K) a single orientation matrix was used due to only a minor splitting of the diffraction peaks. The six runs within each dataset were integrated separately and then combined in ANY to produce a list of integrated intensities of Bragg reflections. The *SADABS* software (Sheldrick, 2008 ▸) was then used for scaling and absorption correction. The Laue class 2/*m* was used in all cases, even for the pseudo-merohedrally twinned phase III of triclinic symmetry (with twin volume fractions constrained to be equal). A preliminary analysis with Laue class 1 led to refined twin volume fractions of 0.5:0.5.

### 2.4. Structural analysis   

*JANA2006* (Petříček *et al.*, 2014 ▸) was used for structure refinements. Initial values of the atomic positions and the modulation parameters were taken from the previously published model in (3+1)-dimensional [(3+1)D] superspace (Rekis *et al.*, 2020 ▸). Crystallographic data of refinements at selected temperatures are listed in Table 1 ▸ (see supporting information for the refinement data at all temperatures).

Hirshfeld surfaces were generated with the software *CrystalExplorer17* (Turner *et al.*, 2017 ▸).

### 2.5. The superspace approach   

The crystal structures of Na(sac)(15/8)H_2_O are described as commensurately modulated structures in (3+1)D superspace, in accordance with our previously published structure model (Rekis *et al.*, 2020 ▸). In comparison with the supercell approach (*b* ≃ 28.4 Å), the superspace approach is based on a small, basic structure unit cell (*b* ≃ 7.1 Å in Table 1 ▸) together with a modulation based on a modulation wavevector **q** = 3/4b* (Table 1 ▸). Atomic positions are determined as the sum of the position in the basic structure and a shift obtained as the value of the modulation function in that particular basic structure unit cell. The periodic modulation functions continuously depend on their arguments as 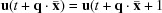), where 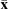 is the position in the basic structure, and *t* is a phase parameter that has one fixed value in each representation of the crystal structure in physical three-dimensional space. For the commensurate modulation of Na(sac)(15/8)H_2_O, eight different values are relevant for the argument 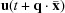. They can be expressed by the eight values *t* = *n*/8 (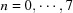). Instead of tables of positions, distances and occupancies, the variations of these quantities throughout the supercell are summarized in *t*-plots of these quantities (van Smaalen, 2012 ▸). For example, Fig. 6 gives the three components of the displacive modulation 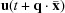 of atom S1a as a function of *t*. In the case of an incommensurate modulation, any value of *t* would be relevant. In the present case of an eightfold superstructure, the values of 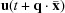 are relevant at the eight values *t* = *n*/8 (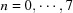), indicated by the vertical dashed lines in Fig. 6.

## 3. Results and discussion   

### 3.1. Structural characterization of Na(sac)(15/8)H_2_O phases   

**Figure 3:**
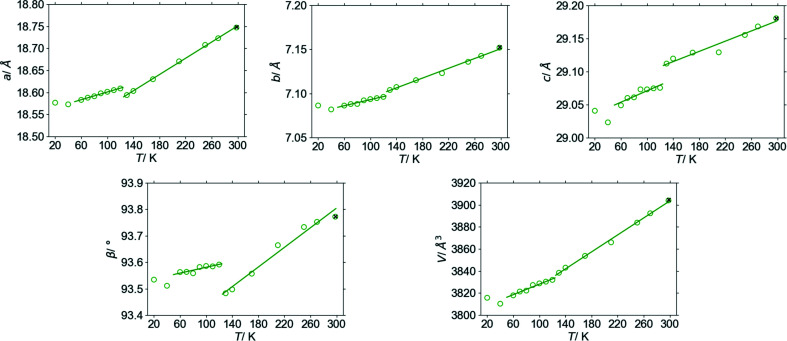
Lattice parameters measured upon cooling the crystal (circles) and after reheating to room temperature (cross). Solid lines represent linear fits to the data within the high-temperature (130 to 298 K) and intermediate (60 to 120 K) phases, respectively.

**Figure 4:**
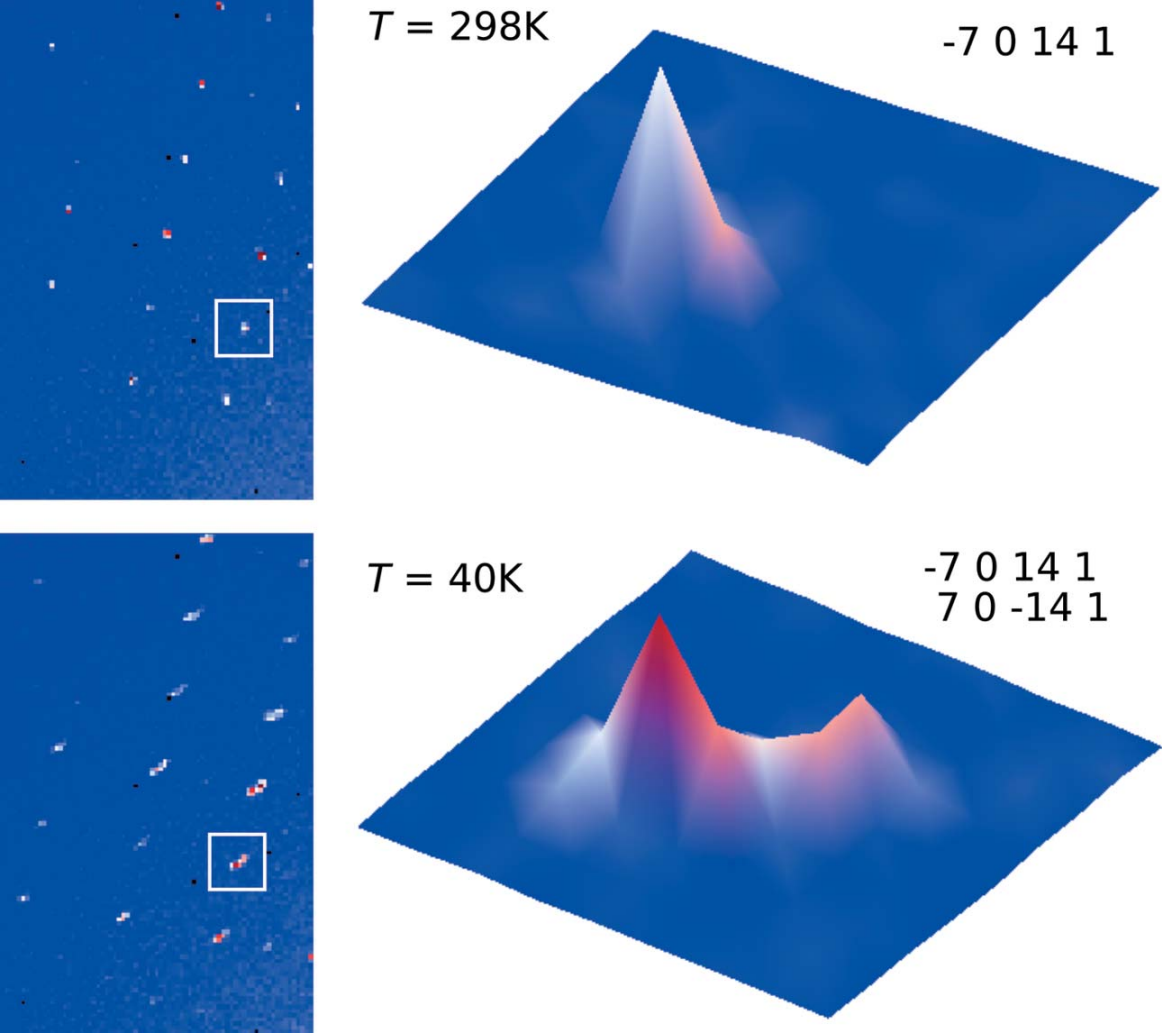
Fragments of the diffraction frames showing reflection −7 0 14 1 at 298 K and the corresponding split reflection at 40 K. Splitting is according to the twin law (−7 0 14 1 and 7 0 −14 1).

Solid-state behaviour of Na(sac)(15/8)H_2_O was explored by measuring a single crystal at selected temperatures in the range from room temperature down to 20 K. Analysis of the lattice parameters and inspection of the diffraction frames revealed that there are two phase transitions, occurring between 130 and 120 K and between 60 and 40 K, respectively. A considerable discontinuity of the unit-cell parameters can be noted at the first transition especially (Fig. 3 ▸). The diffraction maxima are split in the diffraction frames of the 20 and 40 K datasets, indicating the presence of twinning at these temperatures, as opposed to non-split peaks at temperatures above 40 K (Fig. 4 ▸). Twinning indicates a transformation from monoclinic to triclinic symmetries at the second phase transition. Na(sac)(15/8)H_2_O phases will be referred to as phase I (300 to 130 K), phase II (120 to 60 K) and phase III (40 to 20 K). Based on the apparent discontinuity in the lattice parameters as well as in the occupancies of the disordered atoms, it is believed that both transitions are first-order phase transitions. Furthermore, the first-order character of the transition between phases I and II is corroborated by having observed phase I in an undercooled state at 95 K, a feature that is discussed below.

**Figure 5:**
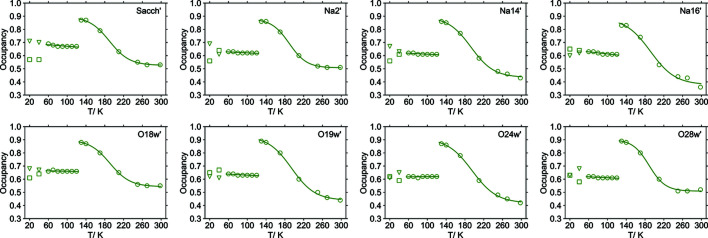
Temperature dependence of the major site occupancy for the entities affected by disorder. Data points (circles) for the high-temperature (130 to 298 K) and intermediate (60 to 120 K) phases have been fitted to sigmoid and linear functions, respectively. Triangles and squares indicate occupancies in the triclinic phase III of sites that have equal occupancies in phases I and II, respectively.

No major structural reorganization can be identified between the refined structure models of the different phases. They are similar to the published structure models, except for features related to the disorder. As a result, the occupancy ratios for each chemical entity were refined separately to explore the disorder in more detail as well as to evaluate how well the correlated disorder assumption actually holds at different temperatures. In Fig. 5 ▸ occupancy values of the disorder components versus temperature are depicted for each of the chemical entities. At room temperature it is approximately 0.5 for each entity (maximum disorder). As the temperature decreases, the occupancy changes in a sigmoid pattern reaching 0.9 for the major components at 130 K (nearly ordered state). Then there is a sudden drop towards occupancies of approximately 0.65 at 120 K. This value stays approximately constant down to 60 K. For the triclinic phase III there is a loss of symmetry, resulting in double the number of crystallographically independent sites in the unit cell.

For some of these pairs of disorder sites there is a clear indication of deviation from monoclinic symmetry (see Fig. 5 ▸).

**Figure 6:**
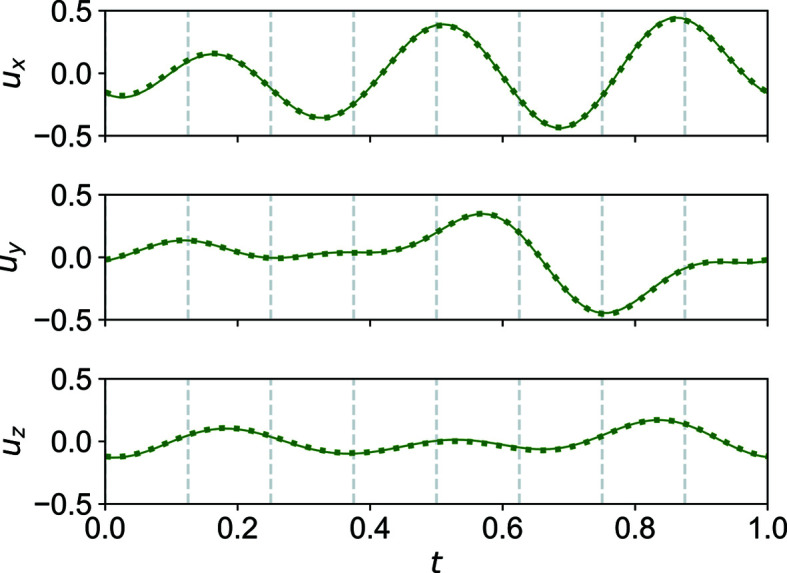
*t*-Plots of the positional modulation of atom S1a (thin solid line: phase I at 210 K; thick dashed line: phase II at 90 K). Commensurate *t*-sections are indicated by vertical dashed lines.

Phases I and II are isomorphous, described in the superspace group *C*2/*c*(0σ_2_0)*s*0 [standard setting *B*2/*b*(00σ_3_)*s*0, No. 15.1.7.3 (Stokes *et al.*, 2011 ▸; van Smaalen *et al.*, 2013 ▸)]. It is indeed intriguing that suddenly the structure becomes more disordered at lower temperature. The disorder of phase II is only weakly dependent on temperature. It corresponds to that of phase I at around 200 K. Apart from the different disorder behaviour the only other difference is the change in the lattice parameters (thermal expansion). It should be noted that the diffraction data and refinements were carefully inspected to look for possible symmetry changes (*e.g.* the loss of inversion symmetry that would result in subgroups *C*2 or *Cc*). Furthermore, no additional disorder was found that would render phases I and II substantially different from each other. No significant changes can be noted comparing relative atomic positions. For example, the modulation functions of atom S1a of phase I at 210 K and of phase II at 90 K are practically overlaid (see Fig. 6 ▸). The same is observed for the other atoms.

**Figure 7:**
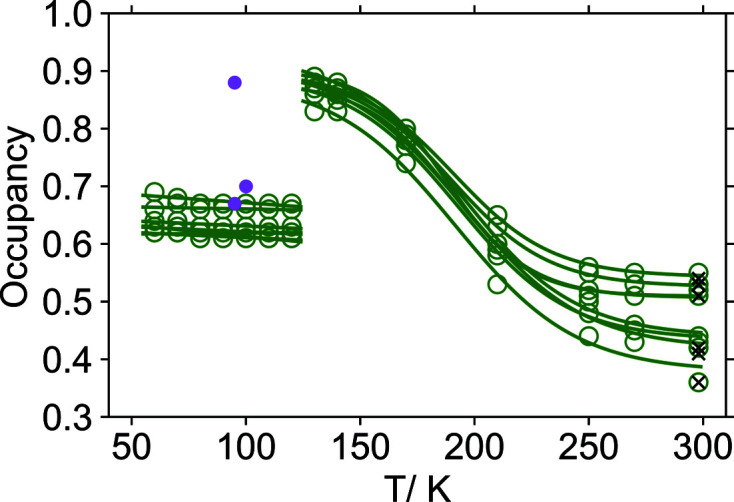
Temperature dependence of the major site occupancy for the entities affected by disorder. Data points (open circles) for the high-temperature (130 to 298 K) and intermediate (60 to 120 K) phases have been fitted to sigmoid and linear functions, respectively. Occupancies from the crystal reheated to room temperature depicted with crosses. Data from previous studies (Naumov *et al.*, 2005 ▸; Banerjee *et al.*, 2005 ▸; Rekis *et al.*, 2020 ▸) are indicated by closed circles.

It is not entirely clear whether the disorder is genuinely correlated at all temperatures. For example, at room temperature the occupancy of the supposedly major (single-primed) disorder component varies between 0.36 (for atom Na16′) and 0.55 (atom O18w′). As the temperature is decreased, the occupancy for phase I tends to a single value. It is most obvious in Fig. 7 ▸ where all data are depicted in a single graph. Observed discrepancies among the disorder ratios of the chemical entities (especially at room temperature) might have a physical significance. Even though in some cases non-correlated disorder might be meaningless (*e.g.* only a single-occupancy ratio is reasonable for the atoms belonging to the saccharinate anions), independent chemical entities can indeed have different occupancy ratios – as long as the numerous resulting spatial organizations are chemically possible (*e.g.* have reasonable interatomic distances). In Table 2 ▸ distances between the disordered atoms in the 298 K structure model are listed. Underlined numbers indicate that most pairs of sites (single-primed and double-primed sites) are too close to each other for both sites to be occupied simultaneously in a single unit cell. Apart from that constraint, which is fulfilled by all structure models, there are only two critically short distances between different sites, namely, between atoms Na14′ and O24w′′ and between atoms Na14′′ and O24w′. Sodium cation–water oxygen atom distances below 2 Å are unlikely to occur in any crystal structure. These short distances imply the disorder Na14′/Na14′′ must be correlated to the disorder O24w′/O24w′′. Indeed, in Table 3 ▸ it is shown that, at room temperature, independently refined occupancies for all atoms have resulted in equal occupancy values for Na14′ and O24w′. At temperatures down to 60 K the corresponding occupancy values do not differ by more than 0.02. It is most likely that the disorder is not fully correlated for all involved chemical entities, except at temperatures around 130 K.

Dynamic disorder effects cannot be excluded, especially close to room temperature. It appears that upon cooling two correlated disorder ensembles emerge with an ever increasing energy gap that leads to a nearly ordered state down to 130 K. Then a phase transition occurs towards increased disorder below 120 K. This reentrant disorder can be understood when the free-energy difference between the two states becomes smaller again. Further cooling has almost no impact on this ratio. In a general case, one would expect that either the disorder becomes frozen-in at a glass transition, or it decreases instead as the energy gap becomes sufficiently large between the two states and the entropy can no longer stabilize a disordered state. In this case, it seems that concerted flips occur, affecting the target occupancy ratio.

In Fig. 7 ▸ the unified occupancy values from the previous structure studies at 95 and 100 K have also been included (Naumov *et al.*, 2005 ▸; Banerjee *et al.*, 2005 ▸; Rekis *et al.*, 2020 ▸). Two of the previous results correspond well to the present data of phase II. The third occupancy value of the major component is the same as for phase I in the vicinity of the phase transition, although it was found for a crystal measured at 95 K (Rekis *et al.*, 2020 ▸), *i.e.* in the region of phase II. We argue that the discrepancy arises because the particular crystal had not gone through the phase transition and thus represents a supercooled state of phase I. Supercooling (or superheating) is a common phenomenon regarding first-order phase transitions. Besides, the particular crystal from the previous study was of exceptionally good quality, thus containing only a few defects that may act as nucleation centres for the intermediate phase, and it was cooled at a rate as low as 2 K min^−1^ (Rekis *et al.*, 2020 ▸), whereas for the present study a rate of 4 K min^−1^ was used. Undoubtedly, slow cooling (or heating) is a common way of obtaining supercooled (or superheated) states, while an excellent crystal lacks defect centres for nucleation of the new phase. There is no information about the cooling procedure of the crystals from previous studies which complies with the present data for phase II.

As mentioned before, there is a second phase transition between 60 and 40 K that leads to a twinned phase (see Fig. 4 ▸ where the characteristic splitting of the peaks is shown). The twinning is pseudo-merohedral – the metric symmetry of phase III is nearly monoclinic, however, the true symmetry is lowered to triclinic. The lost symmetry operator of the monoclinic setting, namely a 180° rotation along b*, acts as a twin operator. Slight deviations of the angles α and γ from 90° (see Table 1 ▸ and the supporting information) lead to the observed small peak splitting that is the very essence of pseudo-merohedral twinning. Intensity statistics indicate that the *C*-centre is retained in phase III. It means that either two twin domains are present [superspace group: *C* 1(σ_1_σ_2_σ_3_)0; standard setting *P* 1(σ_1_σ_2_σ_3_)0, No. 2.1.1.1 (Stokes *et al.*, 2011 ▸)], or four twin domains are present [superspace group: *C*1(σ_1_σ_2_σ_3_)0; standard setting *P*1(σ_1_σ_2_σ_3_)0, No. 1.1.1.1 (Stokes *et al.*, 2011 ▸)]. The latter symmetry adds inversion twinning to each of the two domains. Refinement in *C*1(σ_1_σ_2_σ_3_)0 (four twin domains) diverged considerably, indicating correlated parameters due to missing symmetry. The refinement in *C* 1(σ_1_σ_2_σ_3_)0 (two twin domains) converged.

**Figure 8:**
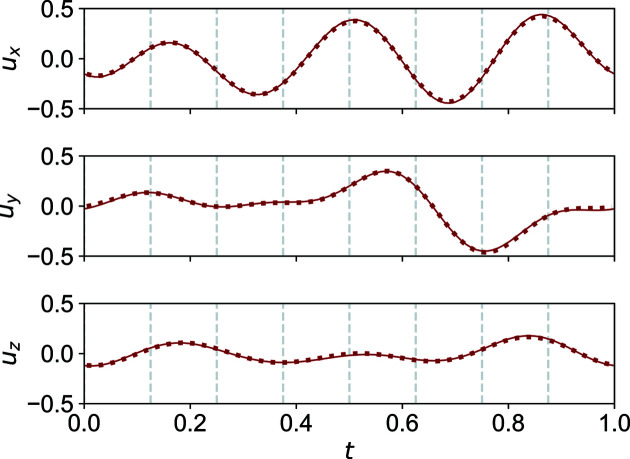
*t*-Plots of the positional modulation of atoms S1a_1 (thin solid line) and S1a_2 (thick dashed line; symmetry transformation applied) in phase III at 40 K. The two atoms would be related by a twofold screw axis in the monoclinic phases I and II. We can see that they do not deviate much from this monoclinic symmetry. Commensurate *t*-sections are indicated by vertical dashed lines.

At the second phase transition, no major reorganization in the structure takes place. In fact, the atomic positions do not deviate much from monoclinic symmetry. For example, in Fig. 8 ▸ the modulation functions are depicted of atom S1a_1 and, symmetry-transformed, of atom S1a_2. The overlay is nearly perfect, indicating a small deviation from monoclinic symmetry in triclinic phase III. Furthermore, the modulation functions do not differ significantly from the corresponding modulation functions in phases I and II (Fig. 6 ▸).

It follows that the largest deviation from monoclinic symmetry, which might induce the particular phase transition towards a twinned triclinic structure, are significant differences in the occupancy ratios for some of the disorder components previously related by symmetry (see Fig. 5 ▸).

After collecting datasets at 20 K the crystal was heated to room temperature and measured again after around 15 h. Both refinements at 298 K correspond very well to each other (see Figs. 7 ▸ and 3 ▸) demonstrating the completely reversible nature of the phase transitions. Even the occupancy values correspond surprisingly well (see Table 3 ▸), once again indicating not fully correlated disorder of the chemical entities at room temperature.

### 3.2. On the microscopic origin of phase transitions in crystalline Na(sac)(15/8)H_2_O   

The transition between phases II and III is not an unusual one as it is rather common for crystals to develop pseudo-merohedral twinning at low temperatures, resulting from lowering of the symmetry and small distortions of some high-temperature crystal structure. The much more intriguing phase transition in this system is the transformation between phases I and II.

As stated above, the structural analysis did not reveal any new chemical contacts nor a major reorganization in the coordination spheres of sodium cations. Nevertheless, minute distortions will be analyzed to better understand the reasons for this unusual phase transition.

**Figure 9:**
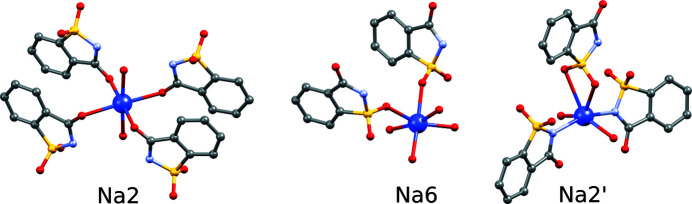
Coordination spheres of selected Na atoms in the structure of Na(sac)(15/8)H_2_O. Sodium cations Na2 and Na6 are octahedrally coordinated by oxygen atoms from saccharinate anions and from water molecules. Sodium atom Na2′ is an isolate (occurring once in the asymmetric unit of the supercell). It demonstrates a special case where the cation is coordinated by four oxygen atoms and two nitrogen atoms from the saccharinate anion.

Most sodium cations are in distorted octahedral coordination to oxygen atoms from water molecules and from saccharinate anions. But this is not true for all cations. The structure of Na(sac)(15/8)H_2_O is extraordinary due to very diverse organization of the sodium cation coordination spheres. In Fig. 9 ▸ examples of several such coordination spheres are shown. In the supercell, there are 16 symmetrically independent sodium cations. Some exhibit similar coordination spheres (as per modulation), like atoms Na2 and Na6, but aside from that some are isolates, like atoms Na14, Na16, Na14′, Na16′, Na2′ (and their disorder component counterparts), with distinct coordination spheres occurring only once in the asymmetric unit.

**Figure 10:**
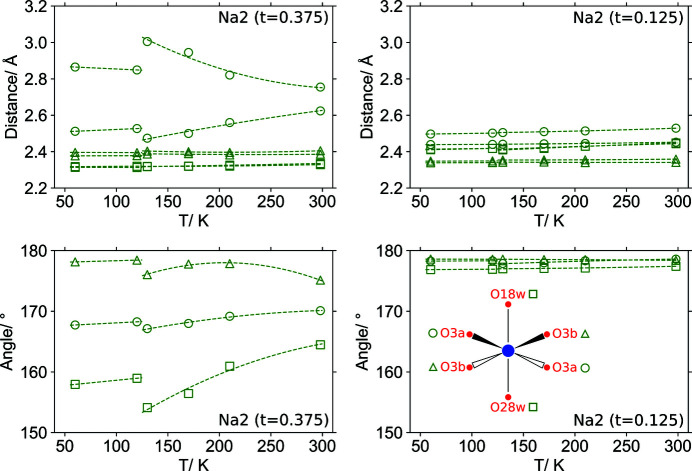
Temperature-dependent evolution of the distortions of the coordination spheres of the sodium cations Na2(*t* = 0.125) and Na2(*t* = 0.375). Distances are given for Na—O contacts and angles for O—Na—O. The geometry of the coordination sphere of cation Na2(*t* = 0.125) does not change significantly upon cooling nor at the phase transition between 130 and 120 K, whereas for cation Na2(*t* = 0.0375) a large distortion occurs involving Na2—O3a contacts.

**Figure 11:**
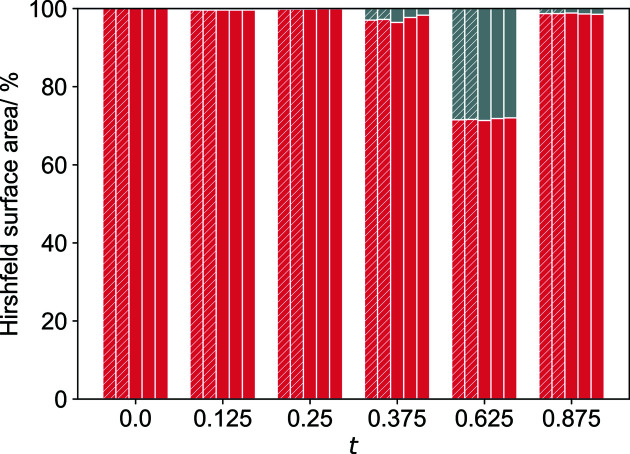
Fraction of contacts defined by Na⋯O (red) and Na⋯*other* (grey) contacts of cations Na2, as obtained from fingerprint plots. The five bars at each *t*-value correspond to structures at different temperatures (from left to right: 60, 120, 130, 210 and 298 K; phase I – regular, phase II – hatched).

There is a temperature-dependent deformation of a number of coordination spheres. Furthermore, very different behaviour can be observed for identically coordinated cations. In Fig. 10 ▸ temperature-dependent evolution of the octahedral distances (Na—O) and angles (O—Na—O) are depicted for atoms Na2(*t* = 0.125) and Na2(*t* = 0.375). In the case of Na2(*t* = 0.125) there is barely any variation within the 60 to 300 K temperature range covering both phases, whereas for atom Na2(*t* = 0.375) the distances to saccharinate O3a atoms change significantly with temperature. These effects are better analyzed on the basis of Hirshfeld surfaces of the atoms (ions), from which the fraction of surface area can be derived for each contact between a central sodium cation and its coordinating atoms. In Fig. 11 ▸, the temperature dependence is depicted for the fractions of the Hirshfeld surfaces constituting Na⋯O and Na⋯*other* interactions for the six Na2 atoms in the supercell. Since Na2 atoms are coordinated by oxygen atoms it is clear that the surface fraction arising from Na⋯O interactions describes energetically efficient interactions that may be considered part of the main stabilizing interactions of this particular crystal structure. For atom Na2(*t* = 0.125) virtually all of the Hirshfeld surface is defined by such interactions, besides it is not temperature-dependent as also indicated by the distance analysis (see Fig. 10 ▸). For atom Na2(*t* = 0.375), the Na⋯O contact fraction in phase I decreases with decreasing temperature, which is consistent with the disproportional increase of the distance to one of the saccharinate O3a atoms as shown in Fig. 10 ▸. Below the phase transition the Hirshfeld surface fraction of Na⋯O contacts is larger again. It can be concluded that the thermal deformation of the lattice upon cooling results in a decrease of energetically efficient intermolecular interactions between the Na2(*t* = 0.375) atom and a saccharinate O3a atom. This is tolerated down to *T* = 130 K, below which the phase transition occurs and a more efficient interaction is restored. The large fraction of Na⋯*other* interactions at *t* = 0.625 are mainly Na⋯N contacts of atom Na2′. For this isolate atom two saccharinate anions are coordinated to the sodium cation by N atoms (Fig. 9 ▸).

The role of the phase transition is surely not attributed to Na2(*t* 0.375) only. In Figs. S1 and S2 of the supporting information Hirshfeld surface analysis graphs are given for all sodium cations as found for structures with single-primed and double-primed disorder components. For nearly all of the sodium cations the Na⋯O contact fraction is constant or increases slightly upon cooling. For a few cations, namely, Na2(*t* 0.375), Na16′ and Na6(*t* = 0.5), it decreases in phase I and is again larger below the phase transition. Those sodium cations could be primarily attributed to the existence of this unusual phase transition between 130 and 120 K.

It is believed that the disorder ratio discrepancy between phases I and II is induced by the discontinuity of unit-cell parameters and interatomic contact distances. In such a way the environments around the disordered entities are changed significantly enough to favour a different equilibrium ratio of the disorder components.

## 4. Conclusions   

The crystal structure of Na(sac)(15/8)H_2_O is commensurately modulated in the temperature range from room temperature down to 20 K. By lowering the temperature the disordered entities tend to prefer one configuration. Besides, it occurs with an increasing concerted fashion leading to fully correlated disorder with the major component occupying close to 90% of sites. The material displays a rare, reversible phase transition between 120 and 130 K. Counter-intuitively, the disorder increases upon cooling through the phase transition. We have argued that the phase transition is induced by 3 out of 16 sodium coordination spheres being distorted upon cooling towards a state of less efficient interactions. This eventually causes a minute reorganization of the structure. This seemingly small reorganization together with the minor changes of the lattice parameters results in a different equilibrium disorder component ratio. Finally, there is a second phase transition towards triclinic symmetry with twinning commonly observed at low temperatures. 

## Supplementary Material

Crystal structure: contains datablock(s) global, 298K_2nd, 298K, 210K, 90K, 40K. DOI: 10.1107/S2052252520015912/lt5033sup1.cif

Experimental details of diffraction experiment at all measured temperatures. DOI: 10.1107/S2052252520015912/lt5033sup2.pdf

Zip of CIFs from diffraction experiments at all measured temperatures. DOI: 10.1107/S2052252520015912/lt5033sup3.zip

CCDC reference: 2048319

## Figures and Tables

**Table 1:** Crystallographic data of selected structure refinements Observed reflections: *F* ^2^ > 3σ(*F* ^2^).

Phase	I	I	I	II	III
*T* (K)	298 (2nd)	298	210	90	40
Crystal data					
Formula	Na(C_7_H_4_NO_3_S)(15/8)H_2_O				
Formula weight (g mol^−1^)	238.94				
Crystal system	Monoclinic	Monoclinic	Monoclinic	Monoclinic	Triclinic
Superspace group	*C*2/*c*(0σ_2_0)*s*0	*C*2/*c*(0σ_2_0)*s*0	*C*2/*c*(0σ_2_0)*s*0	*C*2/*c*(0σ_2_0)*s*0	*C* 1(σ_1_σ_2_σ_3_)0
**q**, *t*_0_	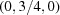 , 0	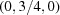 , 0	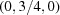 , 0	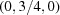 , 0	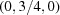 , 0
*a* (Å)	18.74881(2)	18.74705(8)	18.67047(5)	18.59790(4)	18.57290(10)
*b* (Å)	7.15262(4)	7.15189(8)	7.12303(9)	7.09217(5)	7.08190(10)
*c* (Å)	29.18116(4)	29.18066(11)	29.12916(10)	29.07311(5)	29.02330(10)
α (°)	90	90	90	90	89.9808(13)
β (°)	93.77247(5)	93.77227(9)	93.66445(5)	93.58190(6)	93.51100(10)
γ (°)	90	90	90	90	89.9763(14)
*V* (Å^3^)	3904.80(2)	3903.97(4)	3865.98(4)	3827.25(3)	3810.31(6)
*Z*, *Z*′	16, 2	16, 2	16, 2	16, 2	16, 4
*F*(000)	1934	1934	1934	1934	1934
*D_x_* (g cm^−3^)	1.6001	1.6004	1.6161	1.6325	1.6397
μ (mm^−1^)	0.143	0.143	0.144	0.146	0.147
Measured reflections	219920	219580	217545	215114	214582
[sin(θ)/λ]_max_ (Å^−1^)	0.75	0.75	0.75	0.75	0.75
Unique reflections	53543	53580	53016	52326	52280
Observed reflections	23243	26775	30241	35261	34261
*R*_int_ (obs.)	0.0368	0.0388	0.0401	0.0367	0.0657
Refinement					
Refinement method	Full-matrix least-squares on *F*				
No. of parameters	1702	1702	1702	1702	2045
*R*_1_ (obs.)	0.0548	0.0539	0.0518	0.0486	0.0582
*wR* (all)	0.0567	0.0563	0.0544	0.0534	0.0610
GoF (all)	1.74	1.95	2.04	2.32	2.49
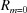 (obs.)	0.0472	0.0498	0.0458	0.0434	0.0544
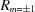 (obs.)	0.0521	0.0504	0.0486	0.0460	0.0564
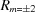 (obs.)	0.0583	0.0565	0.0545	0.0511	0.0597
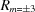 (obs.)	0.0575	0.0557	0.0545	0.0502	0.0595
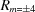 (obs.)	0.0727	0.0677	0.0640	0.0593	0.0655
H-atom treatment	H-atom parameters constrained	H-atom parameters constrained	H-atom parameters constrained	H-atom parameters constrained	H-atom parameters constrained
Weighting scheme	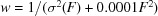				
Δρ_max_ (Å^−3^)	1.10	0.86	1.42	2.18	0.85
Δρ_min_ (Å^−3^)	−1.03	−0.91	−1.34	−1.83	−0.92

**Table 2:** Distances (in Å) between the disordered atoms (298 K model) Distances between the two disordered counterparts are underlined. Critically short distances are depicted in bold.

	sacch (N1b′)	sacch (N1b′′)	sacch (O3b′)	sacch (O3b′′)	Na2′	Na2′′	Na14′	Na14′′	Na16′	Na16′′	O18w′	O18w′′	O19w′	O19w′′	O24w′	O24w′′	O28w′	O28w′′
sacch (N1b′)	0																	
sacch (N1b′′)	0.7	0																
sacch (O3b′)			0															
sacch (O3b′′)			1.1	0														
Na2′	2.7	3.1	4.1	4.9	0													
Na2′′	2.3	2.5	2.8	3.7	1.5	0												
Na14′	4.6	4.3	2.4	2.7	5.8	4.3	0											
Na14′′	4.7	4.0	2.9	2.3	6.0	4.5	3.0	0										
Na16′	5.1	4.8	4.1	4.5	4.6	3.3	3.9	3.5	0									
Na16′′	5.2	5.0	4.1	4.7	4.6	3.4	3.7	3.9	0.8	0								
O18w′	4.2	4.1	2.8	3.7	4.2	2.8	2.3	3.8	2.4	2.0	0							
O18w′′	3.6	3.5	2.3	3.2	3.8	2.4	2.2	3.7	2.8	2.5	0.7	0						
O19w′	3.0	3.0	4.1	4.5	2.4	2.4	6.0	5.0	4.2	4.6	4.9	4.5	0					
O19w′′	3.1	3.2	4.5	5.0	2.1	2.5	6.5	5.6	4.7	5.0	5.2	4.8	0.7	0				
O24w′	5.4	4.8	3.3	2.8	6.8	5.3	2.3	**1.4**	4.1	4.3	3.8	3.8	6.2	6.8	0			
O24w′′	5.6	5.1	3.3	3.0	6.9	5.4	**1.6**	2.3	4.3	4.3	3.5	3.6	6.6	7.2	1.0	0		
O28w′	3.4	3.9	4.0	5.1	2.3	2.3	4.9	6.3	4.9	4.6	3.4	3.0	4.5	4.5	6.7	6.4	0	
O28w′′	3.7	4.2	4.5	5.6	2.0	2.4	5.6	6.7	5.0	4.7	3.9	3.5	4.3	4.3	7.2	7.0	0.8	0

**Table 3:** Refined occupancies of the chemical entities at room temperature in two different measurements

Entity	*T* (K)	
	298	298 (2nd)
Ow18′	0.55	0.54
Ow28′	0.52	0.54
sacch′	0.53	0.53
Na2′	0.51	0.51
Ow19′	0.44	0.42
Na14′	0.43	0.42
Ow24′	0.42	0.41
Na16′	0.36	0.36
