# Supplementary material for: Single-crystal-to-single-crystal phase transitions of commensurately modulated sodium saccharinate 1.875-hydrate
Source: IUCrJ. 2021 Jan 1;8(Pt 1):139–47. doi: 10.1107/S2052252520015912 (PMC7792995; doi:10.1107/S2052252520015912)
Supplement: Supplementary file 2 [file m-08-00139-sup2.pdf]

Single-Crystal to Single-Crystal Phase Transitions of Commensurately Modulated Sodium Saccharinate 1.875-Hydrate

Toms Rekis, Achim M. Schaller, Surya Rohith Kotla, Andreas Schönleber, Leila Noohinejad, Martin Tolkiehn, Carsten Paulmann, Sander van Smaalen*

smash@uni-bayreuth.de

Supporting information

Phase	I	I	I	I	I	I	I	I
T/ K	298[2nd]	298	270	250	210	170	140	130
**Crystal data**								
Formula	$Na(C_7H_4NO_3S) \cdot {}^{15}/_8\, H_2O$							
Formula w.	238.94							
Crystal system	Monoclinic							
Space group	$C2/c(0,\sigma_2,0)s0$							
**q**, t_0	(0, ¾, 0), 0							
a/ Å	18.74881(2)	18.74705(8)	18.72290(9)	18.70776(6)	18.67047(5)	18.62995(7)	18.60326(8)	18.59400(5)
b/ Å	7.15262(4)	7.15189(8)	7.14263(9)	7.13587(8)	7.12303(9)	7.11507(8)	7.10726(6)	7.10397(7)
c/ Å	29.18116(4)	29.18066(11)	29.16831(9)	29.15543(10)	29.12916(10)	29.12876(8)	29.11976(7)	29.11214(5)
β/ °	93.77247(5)	93.77227(9)	93.75256(7)	93.73321(6)	93.66445(5)	93.55680(7)	93.49728(10)	93.48267(6)
V/ Å^3	3904.80(2)	3903.97(4)	3892.34(4)	3883.88(4)	3865.98(4)	3853.68(4)	3842.99(3)	3838.36(3)
Z, Z'	16, 2	16, 2	16, 2	16, 2	16, 2	16, 2	16, 2	16, 2
D_x/ g cm^{-3}	1.6001	1.6004	1.6052	1.6087	1.6141	1.6213	1.6258	1.6278
μ/ mm^{-1}	0.143	0.143	0.144	0.144	0.144	0.145	0.145	0.146
Meas. refl.	219920	219580	218918	218346	217545	216584	215953	216106
$[sin(\theta)/\lambda]_{max}$/ Å^{-1}	0.75							
Unique refl.	53543	53580	53419	53302	53016	52778	52571	52512
Obs. refl.	23243	26775	27512	28963	30241	32872	34556	34771
R_{int} (obs.)	0.0368	0.0388	0.0389	0.0383	0.0401	0.0371	0.0354	0.0353
**Refinement**								
Ref. method	Full-matrix least-squares on F							
No. of param.	1702	1702	1702	1702	1702	1702	1702	1702
R_1 (obs.)	0.0548	0.0539	0.0536	0.0534	0.0518	0.0499	0.0475	0.0471
wR (all)	0.0567	0.0563	0.0559	0.0555	0.0544	0.0530	0.0517	0.0515
GoF (all)	1.74	1.95	1.95	2.03	2.04	2.14	2.21	2.22
$R_{m=0}$ (obs.)	0.0472	0.0498	0.0469	0.0474	0.0458	0.0442	0.0426	0.0426
$R_{m=\pm1}$ (obs.)	0.0521	0.0504	0.0510	0.0496	0.0486	0.0471	0.0450	0.0451
$R_{m=\pm2}$ (obs.)	0.0583	0.0565	0.0561	0.0567	0.0545	0.0521	0.0494	0.0486
$R_{m=\pm3}$ (obs.)	0.0575	0.0557	0.0570	0.0557	0.0545	0.0524	0.0491	0.0488
$R_{m=\pm4}$ (obs.)	0.0727	0.0677	0.0670	0.0681	0.0640	0.0610	0.0573	0.0558
H-atom treatment	constr.	constr.	constr.	constr.	constr.	constr.	constr.	constr.
Weighting sch.	$w = 1 / \sigma^2(F) + 0.0001F^2)$							
$\Delta\rho_{max}$/ e Å^{-3}	1.10	0.86	1.03	1.22	1.42	1.26	1.41	1.17
$\Delta\rho_{min}$/ e Å^{-3}	−1.03	−0.91	−1.01	−0.93	−1.34	−1.56	−1.52	−1.53

Phase	II	II	II	II	II	II	II	III	III
T/ K	120	110	100	90	80	70	60	40	20
**Crystal data**									
Formula	$Na(C_7H_4NO_3S) \cdot {}^{15}/_{8}\ H_2O$								
Formula w.	238.94								
Crystal system	Monoclinic							Triclinic*	
Space group	$C2/c(0,\sigma_2,0)s0$							$C{-}1(\sigma_1,\sigma_2,\sigma_3)0$	
**q**, t_0	(0, ¾, 0), 0							(0, ¾, 0), 0	
a/ Å	18.60894(7)	18.60517(11)	18.60142(5)	18.59790(4)	18.59125(4)	18.58801(6)	18.58299(3)	18.57290(10)	18.57670(10)
b/ Å	7.09612(5)	7.09471(7)	7.09359(7)	7.09217(5)	7.08808(6)	7.08802(8)	7.08624(6)	7.08190(10)	7.08640(10)
c/ Å	29.07549(9)	29.07473(5)	29.07316(10)	29.07311(5)	29.06112(9)	29.06013(7)	29.04898(6)	29.02330(10)	29.04080(10)
β/ °	93.59123(8)	93.58434(12)	93.58574(7)	93.58194(6)	93.55830(5)	93.56348(5)	93.56293(4)	93.51100(10)**	93.53420(10)***
V/ Å^3	3831.92(3)	3830.31	3828.72(3)	3827.24(3)	3822.18(3)	3821.33(4)	3817.88(3)	3810.31(6)	3815.71(6)
Z, Z'	16, 2	16, 2	16, 2	16, 2	16, 2	16, 2	16, 2	16, 4	16, 4
D_x/ g cm^{-3}	1.6305	1.6312	1.6319	1.6325	1.6346	1.635	1.6365	1.6397	1.6374
μ/ mm^{-1}	0.146	0.146	0.146	0.146	0.146	0.146	0.146	0.147	0.146
Meas. refl.	215736	215742	215354	215114	215267	215148	214969	214582	214563
$[sin(\theta)/\lambda]_{max}$/ Å^{-1}	0.75								
Unique refl.	52442	52402	52375	52326	52249	52249	52344	52280	52431
Obs. refl.	34304	34814	34863	35261	35241	35507	36098	34261	34027
R_{int}	0.0360	0.0363	0.0370	0.0367	0.0403	0.0399	0.0367	0.0657	0.0793
**Refinement**									
Ref. method	Full-matrix least-squares on F								
No. of param.	1702	1702	1702	1702	1702	1702	1702	2045	2045
R_1 (obs.)	0.0492	0.0496	0.0491	0.0486	0.0489	0.0492	0.0482	0.0582	0.0665
wR (all)	0.0532	0.0535	0.0533	0.0534	0.0536	0.0537	0.0535	0.0610	0.0695
GoF (all)	2.25	2.29	2.28	2.32	2.31	2.32	2.39	2.49	2.77
$R_{m=0}$ (obs.)	0.0427	0.0439	0.0439	0.0434	0.0438	0.0437	0.0433	0.0544	0.0638
$R_{m=\pm1}$ (obs.)	0.0473	0.0467	0.0466	0.0460	0.0466	0.0467	0.0456	0.0564	0.0643
$R_{m=\pm2}$ (obs.)	0.0521	0.0521	0.0512	0.0511	0.0512	0.0520	0.0500	0.0597	0.0677
$R_{m=\pm3}$ (obs.)	0.0506	0.0518	0.0505	0.0502	0.0503	0.0501	0.0501	0.0595	0.0672
$R_{m=\pm4}$ (obs.)	0.0605	0.0602	0.0602	0.0593	0.0593	0.0605	0.0587	0.0655	0.0740
H-atom treatment	constr.	constr.	constr.	constr.	constr.	constr.	constr.	constr.	constr.
Weighting sch.	$w = 1 / \sigma^2(F) + 0.0001F^2)$								
$\Delta\rho_{max}$/ e Å^{-3}	1.74	1.73	1.89	2.18	1.85	2.02	2.21	0.85	0.98
$\Delta\rho_{min}$/ e Å^{-3}	−1.57	−1.67	−1.80	−1.83	−1.80	−1.76	−1.79	−0.92	−0.91

* – pseudomerohedrally twinned with twin volume fractions virtually 0.5 : 0.5, therefore the data are averaged according to Laue class 2/m and the twin fractions are constrained to exactly 0.5 : 0.5

** – α = 89.9808(13)°; γ = 89.9763(14)°

*** – α = 89.9392(2)°; γ = 89.9939(16)°

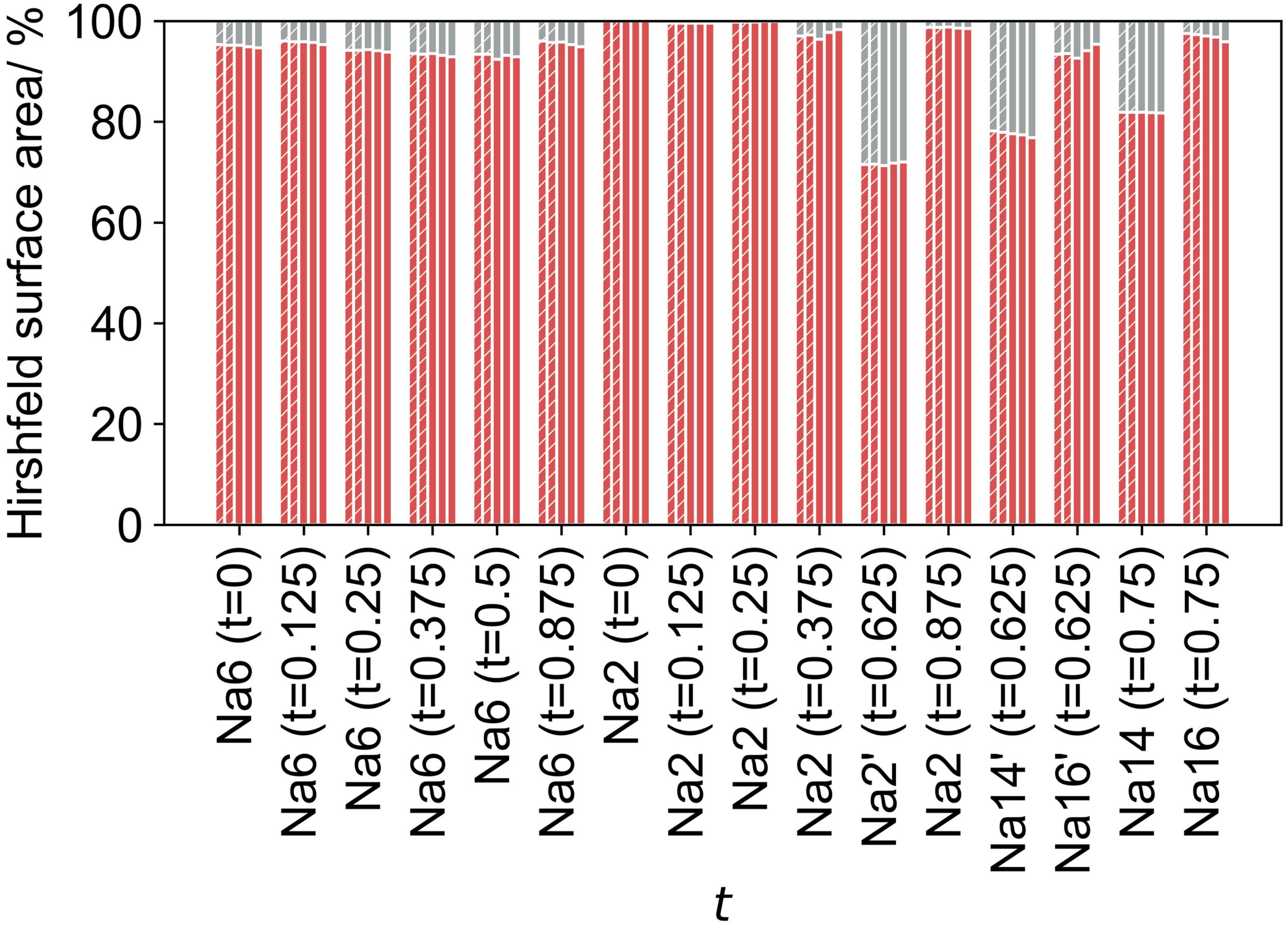

Figure S1. Hirshfeld surface area fractions defined by Na···O (red) and Na···*other* (gray) contacts of cations Na2. The five bars at each *t* value correspond to structures of primed disorder component at different temperatures (from right to left: 298, 210, 130, 120, and 60 K; phase I – regular, phase II – hatched).

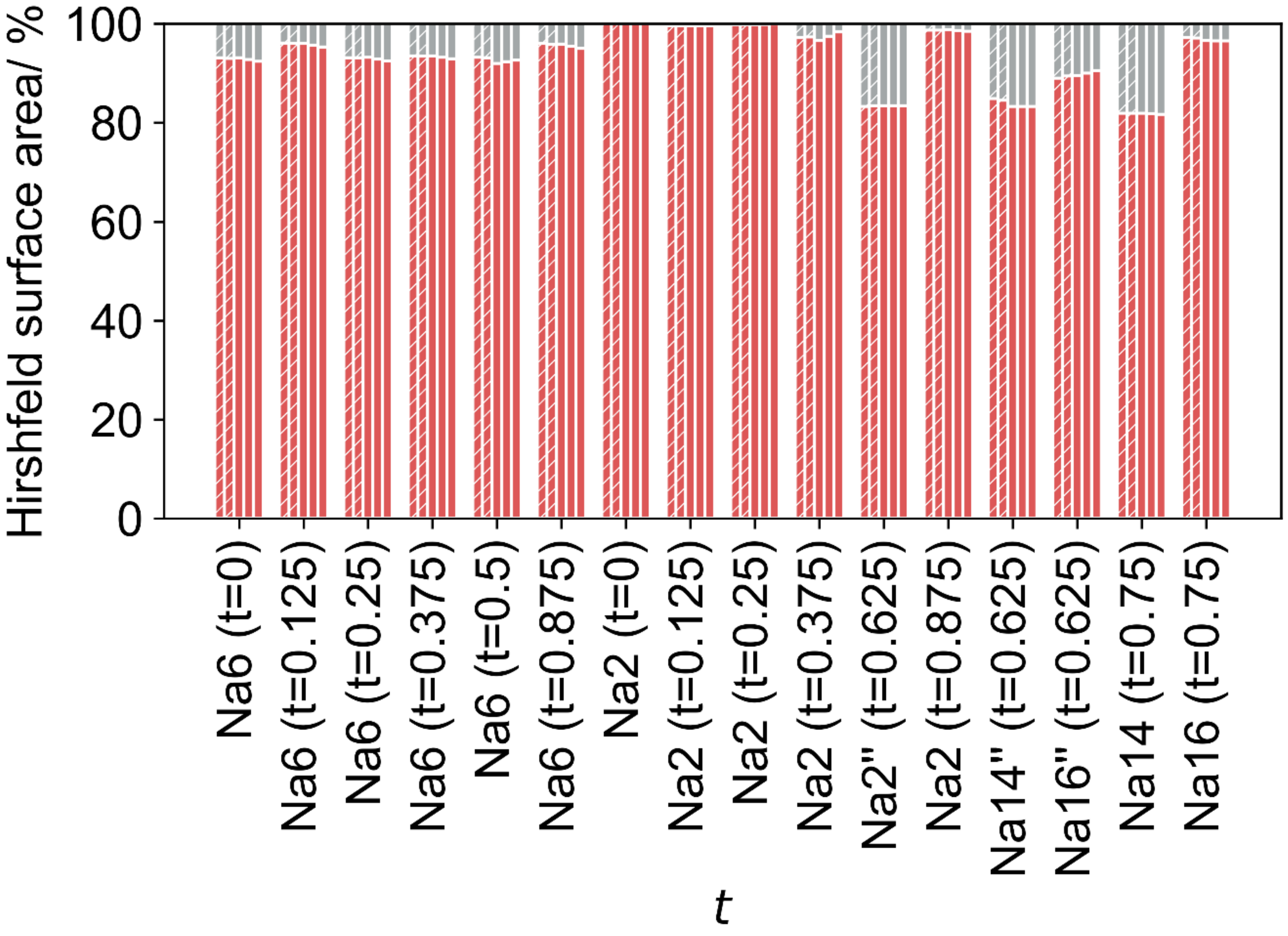

Figure S2. Hirshfeld surface area fractions defined by Na···O (red) and Na···*other* (gray) contacts of cations Na2. The five bars at each *t* value correspond to structures of double-primed disorder component at different temperatures (from right to left: 298, 210, 130, 120, and 60 K; phase I – regular, phase II – hatched).
